# Seasonal Dynamics of Mosquito and Tick Vectors and Molecular Detection of Rift Valley Fever and Crimean–Congo Hemorrhagic Fever Viruses in Transboundary and Non-Transboundary Areas of Senegal

**DOI:** 10.3390/tropicalmed11070173

**Published:** 2026-06-24

**Authors:** Thialao Sarr, Mame Thierno Bakhoum, Aminata Ba, Gorgui Diouf, Moussa Fall, Mamadou Lamine Djiba, Abdou Samath Thiall, Modou Moustapha Lo, Jessica Radzio Basu, Assane Gueye Fall

**Affiliations:** 1 Laboratoire National de l’Élevage et de Recherches Vétérinaires (ISRA/LNERV), Institut Sénégalais de Recherches Agricoles, Dakar BP 2057, Senegal; baaminata10@gmail.com (A.B.); dgorgui88@gmail.com (G.D.); moussafall08@yahoo.fr (M.F.); malaminedjiba@gmail.com (M.L.D.); abdousamaththiall@gmail.com (A.S.T.); moustaphlo@yahoo.fr (M.M.L.); assane.fall@isra.sn (A.G.F.); 2 The Huck Institute of the Life Sciences, Pennsylvania State University, State College, PA 16802, USA; jradzio@gmail.com

**Keywords:** rift valley fever virus, Crimean–Congo hemorrhagic fever virus, vector surveillance, mosquitoes, ticks, seasonality, Senegal

## Abstract

Rift Valley fever virus (RVFV) and Crimean–Congo hemorrhagic fever virus (CCHFV) are endemic zoonotic pathogens in Senegal, transmitted by mosquitoes and ticks, respectively. Understanding the seasonal and spatial dynamics of their vectors is essential to improve targeted surveillance. This study investigated the abundance, diversity, and viral infection status of vector populations in a transboundary region (Matam) and a non-transboundary region (Thiès) over two seasons from September 2022 to March 2024. We collected mosquitoes using CO_2_-baited CDC light traps and sampled ticks directly from domestic small ruminants. A total of 6558 mosquitoes across 23 species and 1904 ticks representing seven species were morphologically identified. Mosquito abundance peaked significantly during the rainy season. Conversely, tick diversity increased during the dry season, with *Hyalomma rufipes* emerging as the predominant species. Crucially, RVFV was detected exclusively in *Aedes vexans* mosquito pools from the transboundary Matam region, emphasizing the epidemiological risk associated with cross-border livestock mobility. Viral RNA of CCHFV was detected in multiple tick species across both regions and seasons, confirming a sustained, multi-vector enzootic cycle. These findings demonstrate persistent RVFV and CCHFV circulation in Senegal and highlight the critical need for integrated, season-specific vector surveillance frameworks.

## 1. Introduction

Rift Valley fever virus (RVFV) and Crimean–Congo hemorrhagic fever virus (CCHFV) are priority zoonotic arboviruses causing substantial public health and veterinary impacts across Africa. In Senegal, both viruses circulate enzootically, triggering recurrent outbreaks linked to varied ecological and anthropogenic drivers. CCHFV, the causative agent of Crimean–Congo hemorrhagic fever (CCHF), is a broadly distributed zoonotic pathogen endemic to regions across Africa, Europe, and Asia [1,2]. Viral transmission to humans is primarily driven by bites from infected *Hyalomma* ticks, which serve as the principal vectors of CCHFV, or through direct exposure to the blood, tissues, and bodily fluids of viremic animals or patients [3,4,5]. Consequently, occupational cohorts with high exposure to livestock or clinical cases, such as livestock farmers, veterinarians, abattoir staff, and healthcare personnels, constitute the highest-risk demographic [6,7]. The case fatality rate among hospitalized patients ranges from approximately 5% to 30% [8]. In Senegal, human infections have been documented across multiple geographic zones [9,10], including instances of cross-border viral importation from Mauritania [11]. The sustained endemic circulation of CCHFV in Senegal is strongly supported by both serological and entomological surveillance; current data demonstrate a seroprevalence of 0.7% in human population and 14.1% in domestic small ruminants, which is further corroborated by the recurrent by molecular detection of CCHFV in tick populations sampled from diverse ecological landscapes nationwide [12,13,14].

RVFV, the causative agent of Rift Valley fever (RVF), is a critical zoonotic pathogen that inflicts severe veterinary and socio-economic impacts [15,16]. The virus remains endemic in Senegal, driving periodic outbreaks in livestock and humans, especially within the northern and central territories [17,18]. Human infections generally present as an undifferentiated febrile illness that complicates clinical diagnosis, though severe cases may escalate to hemorrhagic fever or encephalitis [19]. In domestic ruminants, however, RVFV is highly pathogenic, routinely triggering catastrophic abortion storms and high neonatal mortality that disrupt local agricultural economies [17,20]. In West Africa, the principal mosquito species facilitating viral transmission are vectors include *Aedes vexans*, *Aedes ochraceus*, and *Culex poicilipes* [18,21].

RVF activity in Senegal has notably intensified in recent years. In September 2025, a major outbreak involving both human and animal populations was declared, primarily affecting northern livestock-producing regions such as Senegal River valley and transboundary borders with Mauritania [22]. This outbreak emerged following extensive rainfall and flooding, environmental conditions known to drive massive mosquito proliferation and RVFV transmission. The transmission cycle was further amplified by localized livestock movements and pastoral transhumance. Such events emphasize Senegal’s enduring vulnerability to RVF re-emergence and reinforce the urgent need to comprehensively monitor seasonal vector dynamics in these vulnerable settings.

Transboundary regions of Senegal, particularly those sharing borders with Mauritania and Mali, are characterized by intense livestock movements and seasonal pastoral transhumance. These anthropogenic and ecological dynamics hypothesized to play a critical role in the enzootic maintenance and geographic expansion of both RVFV and CCHFV by altering host availability, vector distribution, and active virus circulation [12,13,17]. Despite this well-recognized epidemiological risk, comparative data evaluating the seasonal dynamics, relative abundance, and ecological community structure of mosquito and tick vectors between transboundary and non-transboundary areas of Senegal remain notably scarce.

Addressing these critical knowledge gaps, the present study aimed to evaluate the seasonal dynamics of vector populations driving RVFV and CCHFV transmission by comparing a transboundary area (Matam) with a non-transboundary area (Thiès). Specifically, our primary objectives were to (i) characterize the species composition, relative abundance, and ecological community structure of mosquitoes and ticks across disparate seasons, and (ii) conduct targeted molecular detection to determine the viral infection status of RVFV and CCHFV within key vector species.

## 2. Materials and Methods

### 2.1. Study Areas

**Figure 1:**
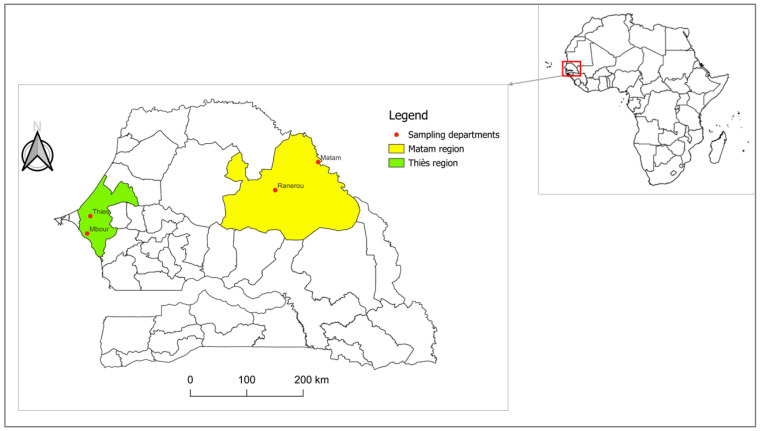
Map showing tick and mosquito sampling sites in Senegal.

The study was conducted in two ecologically contrasting regions of Senegal: the transboundary region of Matam and the non-transboundary region of Thiès (Figure 1).

The Matam region (Transboundary), located in northeastern Senegal bordering Mauritania and Mali, features a semi-arid Sahelian climate with a single rainy season (May to October) and annual precipitation between 300 and 800 mm, creating highly productive, short-lived breeding sites ideal for floodwater *Aedes* mosquitoes. Livestock production relies heavily on extensive, semi-intensive, and mixed farming systems. Epidemiologically, the area is heavily influenced by intense seasonal transhumance involving both national and transboundary herds.

The non-transboundary Thiès region located in western Senegal near the Cape Verde Peninsula, features coastal and peri-urban environments. It experiences a rainy season from June to October with a mean annual precipitation of approximately 360 mm, and is defined by high human population density and intensive livestock production. The region’s hydrologically stable, anthropogenically modified wetlands (e.g., permanent drainage canals and agricultural reservoirs) actively drive the continuous breeding of *Culex* species.

### 2.2. Entomological Sampling

Entomological sampling was conducted across 15 localities within four departments: Matam and Ranérou (transboundary Matam region), and Mbour and Thiès (non-transboundary Thiès region). Field collections spanned both rainy and dry seasons. Sampling occurred over four sessions (two per season) between September 2022 and March 2024. Mosquitoes were collected using CO_2_-baited CDC light traps operated overnight (6 pm to 6 am) for two consecutive nights. At each site, two traps were deployed: one near livestock enclosures and one close to natural water bodies such as rivers, ponds, or lakes. Ticks were collected directly from small domestic ruminants (sheep and goats) across various attachment sites to preserve specimen integrity. Tick attachment sites included the ears, head and neck, upper body, dewlap and abdomen, and genital region, tail, and feet. All collected specimens were placed in labeled 15 mL tubes and transported on dry ice to the laboratory. Morphological identification was performed using a stereomicroscope and standard taxonomic keys for mosquitoes [23,24] and ticks [25].

### 2.3. Pooling Strategy and Storage

After identification, specimens were sorted and pooled to optimize molecular detection sensitivity while minimizing PCR inhibition. Mosquitoes were grouped by species into pools of ≤30 individuals, based on collection site and date. Because of their larger tissue volume, ticks were pooled monospecifically into smaller groups of ≤5 individuals based on collection site, host animal, and attachment site. All pools were stored at −80 °C prior to molecular processing.

### 2.4. Molecular Detection of RVFV and CCHFV

Total RNA was extracted using the Direct-zol™ RNA MiniPrep kit (Zymo Research, Irvine, CA, USA) following the manufacturer’s instructions. The detection of the viral genomes was performed via real time RT-PCR assays using the Light Mix^®^ + Primers/Probe System kit (TIB MOLBIOL distrivuter: Roche Diagnostics, Berlin, Germany). Specific primers and probes, formulated by the Pasteur Institute of Dakar for the COHWA (Coordinated One Health approach to risk assessment of hemorrhagic fever viruses in West Africa) consortium (Table A1), were used to target RVFV (mosquito pools) and CCHFV (tick pools). Samples yielding a distinct exponential amplification curve with a cycle threshold (Ct) value ≤ 35 were classified as positive.

### 2.5. Data Analysis

Statistical analyses were conducted using R software v4.3.1. Species accumulation curves, species richness, the Shannon diversity index (H), apparent density (AD), and the Berger–Parker dominance index were calculated for each region and season. To assess the significance of spatiotemporal variations, non-parametric comparative tests (Kruskal–Wallis) for abundance were applied across regions and seasons.

## 3. Results

### 3.1. Seasonal Abundance and Diversity of Mosquitoes

**Figure 2:**
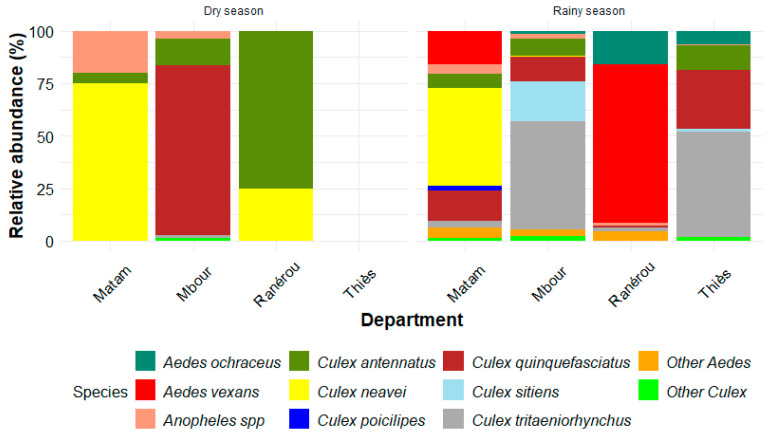
Variation in the relative abundance of Mosquito species in different departments: Matam and Ranérou (transboundary region), and Mbour (non-transboundary region).

A total of 6558 mosquitoes belonging to 23 species were collected. Abundance was markedly higher during the rainy season (6456 individuals) compared with the dry season (102 individuals) (*p*-value = 2.073 × 10^−6^) (Table A2, Table 1 and Figure 2). *Aedes vexans* and *Aedes ochraceus* predominated in the transboundary region (Matam), while *Culex quinquefasciatus* and *Culex tritaeniorhynchus* dominated the coastal and peri-urban environments of the non-transboundary region (Thiès) (Table 2).

The highest apparent densities were observed in Thiès and Mbour during the rainy season, whereas Matam and Ranérou showed lower but variable abundances (Table 1). Species diversity peaked during the rainy season, with Matam exhibiting the highest Shannon diversity index (H = 1.98) and Ranérou the lowest (H = 0.85). During the dry season, mosquito abundance and diversity declined substantially, with some sites, particularly Thiès, showing near absence of mosquitoes (Table 1).

### 3.2. Seasonal Abundance and Diversity of Ticks

**Figure 3:**
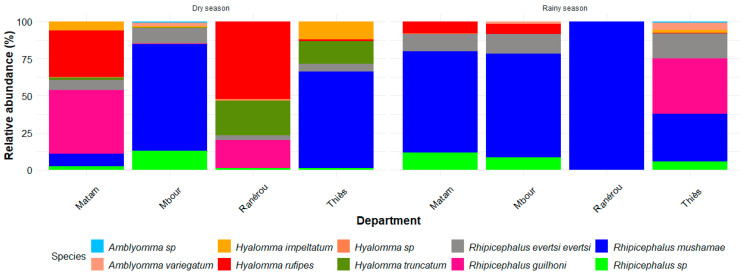
Variation in the relative abundance of tick species in different departments: Matam and Ranérou (transboundary region), and Mbour (non-transboundary region).

A total of 1904 ticks comprising seven species were recovered from 587 small ruminants (Table A3). While overall tick abundance remained temporally stable across seasons (no significant difference, *p*-value = 0.2322), the species composition shifted significantly. Tick abundance was relatively similar between rainy and dry seasons, but species composition varied seasonally (Table 3, Figure 3). During the dry season, species richness increased across all survey sites, with Hyalomma rufipes emerging as the overwhelmingly dominant species in the transboundary region (Table 4).

### 3.3. Molecular Identification of RVFV and CCHFV in Arthropod Vectors

Molecular analysis revealed the presence of RVFV RNA in six mosquito pools, all of which originated exclusively from the transboundary region of Matam. Notably, these RVFV-positive pools comprise entirely of *Aedes vexans* specimens collected during the rainy season.

CCHFV RNA was detected in 35 tick pools distributed across both study regions and sampling seasons. This widespread detection encompassed a diverse array of vector species, specifically *Hyalomma rufipes*, *Hyalomma impeltatum*, *Rhipicephalus evertsi evertsi*, *Rhipicephalus guilhoni*, and *Rhipicephalus mushamae* (Table 5).

## 4. Discussion

This study provides a critical comparative evaluation of the seasonal dynamics of RVFV and CCHFV vectors across transboundary (Matam and Ranérou departments) and a non-transboundary (Mbour and Thiès departments) regions of Senegal. By pairing entomological surveillance with molecular screening across two seasons, we demonstrate how rainfall, landscape features, and livestock mobility converge to dictate arboviral transmission risk. This comparative study yields two critical epidemiological insights. First, RVFV was exclusively detected in *Aedes vexans* mosquitoes within the transboundary region during the rainy season. Second, CCHFV actively circulates year-round across multiple tick species regardless of transboundary status, highlighting a complex maintenance cycle.

Seasonal and spatial patterns of RVFV mosquito vectors: Mosquito abundance and diversity were highly seasonal, peaking sharply during the rainy season and emphasizing the reliance of RVFV vector emergence on rainfall and temporary aquatic habitats. This aligns with previous studies demonstrating that rainfall-driven pond dynamics govern the proliferation of *Aedes* and *Culex* mosquitoes and subsequent RVFV transmission [21,26,27,28,29]. Spatially, clear ecological contrasts emerged. The transboundary departments (Matam and Ranérou) in the transboundary region were dominated by the primary RVFV vectors, *Aedes vexans* and *Aedes ochraceus*. In contrast, the non-transboundary departments (Thiès and Mbour) supported higher overall mosquito densities with a broader representation of *Culex* species, including *Culex tritaeniorhynchus* and *Cx. quinquefasciatus*.

Diversity indices highlighted further intra-regional differences. The department of Ranérou (transboundary region) exhibited high mosquito abundance but low Shannon diversity, driven by the overwhelming dominance of *Ae. vexans*. Conversely, the department of Matam (transboundary region) maintained a more balanced mosquito community, characterized by greater diversity despite moderate abundance. Such dominance patterns are epidemiologically critical; environments dominated by highly competent vectors can sustain elevated transmission risks even when overall mosquito populations are moderate [30,31]. During the dry season, mosquito populations collapsed across all sites. The department of Thiès (non-transboundary region) became almost entirely devoid of mosquitoes due to the evaporation of surface water, underscoring the absolute dependence of these vectors on seasonal hydrology. A major finding of this study is the exclusive molecular detection of RVFV in mosquito pools from the transboundary region. Specifically, all RVFV-positive pools consisted entirely of *Aedes vexans*, confirming its central role in RVFV maintenance and early transmission. This underscores the epidemiological importance of transboundary areas, where intense livestock movement and transhumance along the Senegal–Mauritania corridor likely drive viral introduction and amplification. Previous investigations have similarly linked recurrent RVF outbreaks in northern Senegal to animal mobility and transboundary trade, identifying the region as a high-risk zone [18,32,33]. Our entomological and molecular data strongly support these conclusions, highlighting the critical need for targeted surveillance in these transboundary settings.

Tick Ecology and Multi-Vector CCHFV Circulation: Unlike mosquitoes, tick populations maintained relatively stable abundances across seasons but displayed distinct shifts in species dominance. *Hyalomma rufipes*, a primary CCHFV vector highly adapted to arid conditions, dominated during the dry season. This pattern aligns with previous studies indicating that *Hyalomma* species are well adapted to arid and semi-arid environments and often peak during warmer, drier periods when conditions favor their activity and reproduction [34,35]. The detection of CCHFV in multiple tick species including *H. rufipes*, *H. impeltatum*, *Rhipicephalus evertsi evertsi*, *R. guilhoni*, and *R. mushamae* across both seasons and regions demonstrates sustained enzootic circulation of the virus in Senegal. While *Hyalomma* ticks are considered the principal vectors due to their life cycle involving both small vertebrates and large livestock, the repeated detection of viral RNA in *Rhipicephalus* species suggests that CCHFV maintenance may involve a more complex multi-vector system. Similar findings have been reported in Senegal, indicating that *Rhipicephalus* ticks may contribute to local virus persistence, particularly in areas where *Hyalomma* densities are low [12,36].

Eco-Epidemiological Implications and Future Directions for RVFV and CCHFV Surveillance: The concurrent detection of RVFV and CCHFV confirms a persistent zoonotic risk across both study regions. RVFV transmission aligns with a recognized two-stage model, initiated by floodwater *Aedes* mosquitoes and subsequently amplified by *Culex* species, whereby vertically infected *Aedes* mosquitoes initiate transmission following pond flooding, followed by amplification through Culex species in more stable aquatic habitats [27,37]. Conversely, the year-round presence of CCHFV in ticks creates a continuous exposure risk for farmers, herders, veterinarians, slaughterhouse workers, and healthcare personnel [1,3]. A major strength of this study lies in its integrated design, combining mosquito and tick surveillance across transboundary and non-transboundary areas over two seasons, with molecular confirmation of virus circulation. However, the study did not explicitly incorporate environmental variables such as hydrological dynamics, land use, or vegetation cover. Future research integrating satellite-derived indicators (e.g., hydroperiod and NDVI) with entomological and tick data could further improve risk prediction models, as demonstrated in previous eco-epidemiological studies in northern Senegal [27,29].

Implications for surveillance and control: Overall, these findings dictate a prioritized, season-specific One Health intervention strategy. RVFV prevention must focus on intensive mosquito surveillance at the onset of the rainy season. Concurrently, reducing CCHFV transmission requires tick control (e.g., acaricide rotation) and heightened biosafety protocols for herders and abattoir workers during the dry season, accounting for the multi-vector capacity of both *Hyalomma* and *Rhipicephalus* ticks.

## 5. Conclusions

This study provides a comprehensive comparative assessment of the seasonal dynamics of mosquito and tick vectors associated with Rift Valley fever virus (RVFV) and Crimean–Congo hemorrhagic fever virus (CCHFV) in Senegal. By contrasting transboundary (Matam and Ranérou) and non-transboundary region (Mbour and Thiès) regions, we demonstrate that mosquito populations are subject to pronounced seasonal fluctuations, characterized by peak abundance and diversity during the rainy season, due to the availability of rainfall-dependent breeding habitats. Notably, *Aedes* species predominated in inland regions, and RVFV was detected exclusively in mosquito pools from the transboundary region, underscoring the critical epidemiological role of cross-border livestock movements. In contrast, tick populations exhibited less pronounced seasonality but clear shifts in species dominance, with *Hyalomma rufipes* prominent during the dry season. The detection of CCHFV in multiple tick species across regions confirms sustained enzootic circulation and suggests a multi-vector transmission system. Overall, these findings underscore the need for season- and region-specific, One Health-based surveillance and control strategies to reduce the risk of RVF and CCHF in Senegal.

## Figures and Tables

**Table 1:** Diversity of mosquitoes in the different departments of transboundary region and non-transboundary region.

Season	Diversity	Transboundary Region		Non-Transboundary Region	
		Matam	Ranérou	Mbour	Thiès
Rainy	AD (sp/cs/d) *	9.30	22.79	21.00	59.33
	Specific richness observed (S)	22	10	18	14
	Shannon index (H)	1.98	0.85	1.55	1.31
	Specific richness estimated (NS)	22 ± 0.24	10 ± 0.23	18 ± 0.24	14.75 ± 1.41
Dry	AD (sp/cs/d)	1.54	1.33	3.90	0.00
	Specific richness observed (S)	3	2	6	0
	Shannon index (H)	0.68	0.56	0.69	0.00
	Specific richness estimated (NS)	3 ± 0.41	2 ± 0.35	7.5 ± 2.54	NA

*: AD = Apparent density; sp = Specimens; cs = Collection site; d = day.

**Table 2:** Composition and dominance of mosquito species in each department of transboundary (Matam and Ranérou) and non-transboundary (Mbour and Thiès) regions. The index d value of the dominant species is marked in bold.

		Dominance Index (d)			
Season	Species	Matam	Ranérou	Mbour	Thiès
Rainy	*Aedes aegypti*	0.0136	0.0053	0.0291	0.0004
	*Aedes africanus*	0.0031	0.0000	0.0000	0.0000
	*Aedes argenteopunctatus*	0.0000	0.0000	0.0000	0.0008
	*Aedes mcintoshi*	0.0000	0.0319	0.0000	0.0000
	*Aedes metallicus*	0.0021	0.0000	0.0000	0.0004
	*Aedes ochraceus*	0.0031	**0.1582**	0.0156	0.0649
	*Aedes* spp.	0.0125	0.0000	0.0000	0.0008
	*Aedes sudanensis*	0.0177	0.0053	0.0034	0.0000
	*Aedes vexans*	**0.1472**	**0.7580**	0.0013	0.0000
	*Anopheles gambiae*	0.0094	0.0000	0.0097	0.0004
	*Anopheles pharoensis*	0.0084	0.0000	0.0025	0.0000
	*Anopheles rufipes*	0.0157	0.0000	0.0004	0.0000
	*Anopheles* spp.	0.0052	0.0093	0.0063	0.0021
	*Anopheles squamosus*	0.0010	0.0000	0.0000	0.0008
	*Anopheles ziemanni*	0.0042	0.0013	0.0021	0.0000
	*Culex antennatus*	0.0647	0.0000	0.0796	**0.1184**
	*Culex bitaeniorhynchus*	0.0000	0.0000	0.0000	0.0135
	*Culex decens*	0.0000	0.0000	0.0046	0.0038
	*Culex ethiopicus*	0.0261	0.0000	0.0000	0.0000
	*Culex neavei*	**0.4384**	0.0027	0.0055	0.0000
	*Culex poicilipes*	0.0198	0.0000	0.0008	0.0000
	*Culex quinquefasciatus*	**0.1357**	0.0093	**0.1155**	**0.2823**
	*Culex sitiens*	0.0073	0.0000	**0.1926**	0.0126
	*Culex* spp.	0.0104	0.0000	0.0156	0.0000
	*Culex tritaeniorhynchus*	0.0292	0.0186	**0.5120**	**0.4985**
	*Culex univittatus*	0.0251	0.0000	0.0034	0.0000
Dry	*Anopheles gambiae*	0	0	0.01	0
	*Anopheles ziemanni*	**0.2**	0	0.03	0
	*Culex antennatus*	0.05	**0.75**	**0.13**	0
	*Culex neavei*	**0.75**	**0.25**	0	0
	*Culex quinquefasciatus*	0	0	**0.81**	0
	*Culex* spp.	0	0	0.01	0
	*Culex tritaeniorhynchus*	0	0	0.01	0

**Table 3:** Diversity of ticks in the different departments of the transboundary region and the non-transboundary region.

Season	Diversity	Transboundary Region		Non-Transboundary Region	
		Matam	Ranérou	Mbour	Thiès
Rainy	AD (sp/cs/d) *	2.58	1.16	1.46	2.13
	Specific richness observed (S)	5	1	5	8
	Shannon index (H)	0.97	0.00	0.97	1.47
	Specific richness estimated (NS)	5 ± 0.44	1 ± 0.00	5 ± 0.44	8 ± 0.46
Dry	AD (sp/cs/d)	2.14	1.76	2.55	2.33
	Specific richness observed (S)	8	6	8	6
	Shannon index (H)	1.46	0.94	1.21	1.08
	Specific richness estimated (NS)	8 ± 0.00	7 ± 2.22	8 ± 0.91	6 ± 0.00

*: AD = Apparent density; sp = Specimens; cs = Collection site; d = day.

**Table 4:** Composition and dominance of tick species in each department of transboundary (Matam and Ranérou) and non-transboundary (Mbour and Thiès) regions. The index d value of the dominant species is marked in bold.

Season	Species	Matam	Ranérou	Mbour	Thiès
Rainy	*Amblyomma* spp.	0	0	0	0.0067
	*Amblyomma variegatum*	0	0	0.0167	0.0503
	*Hyalomma impeltatum*	0	0	0	0.0201
	*Hyalomma rufipes*	0.0811	0	0.0667	0.0017
	*Hyalomma* spp.	0.0030	0	0	0
	*Rhipicephalus evertsi evertsi*	**0.1171**	0	**0.1333**	**0.1695**
	*Rhipicephalus guilhoni*	0	0	0	**0.3758**
	*Rhipicephalus mushamae*	**0.6847**	**1**	**0.7**	**0.3188**
	*Rhipicephalus* spp.	**0.1141**	0	0.0833	0.0570
Dry	*Amblyomma* spp.	0	0.00	0.0082	0
	*Amblyomma variegatum*	0	0	0.0245	0
	*Hyalomma impeltatum*	0.0621	0	0.0082	**0.1190**
	*Hyalomma rufipes*	**0.3105**	**0.5222**	0	0.0119
	*Hyalomma* spp.	0.0065	0.0111	0	0
	*Hyalomma truncatum*	0.0131	**0.2333**	0.0041	**0.1548**
	*Rhipicephalus evertsi evertsi*	0.0686	0.0333	**0.1020**	0.0516
	*Rhipicephalus guilhoni*	**0.4314**	**0.1889**	0.0041	0
	*Rhipicephalus mushamae*	0.0850	0	**0.7224**	**0.6508**
	*Rhipicephalus* spp.	0.0229	0.0111	**0.1265**	0.0119

**Table 5:** Molecular detection of CCHF virus in tick pools within the transboundary and the non-transboundary regions.

Tick Species	Transboundary Region		Non-Transboundary Region	
	Dry Season	Rainy Season	Dry Season	Rainy Season
*Hyalomma impeltatum*	1	-	-	-
*Hyalomma rufipes*	2	4	-	-
*Rhipicephalus evertsi evertsi*	1	-	2	1
*Rhipicephalus guilhoni*	6	3	-	4
*Rhipicephalus mushamae*	-	9	1	1

## Data Availability

Data supporting the findings of this study are available from the corresponding author upon reasonable request.

## Abbreviations

The following abbreviations are used in this manuscript:

RVFV	Rift Valley fever virus
CCHFV	Crimean–Congo hemorrhagic fever virus
CDC	Centers for Disease Control and Prevention
RT-PCR	Reverse Transcription Polymerase Chain Reaction

## Appendix A

**Table A1:** Primers and probes used for molecular detection.

Pathogens	Primers and Probes	Primer and Probe Sequences
RVFV	RVF_FP	TGCCACGAGTYAGAGCCA
	RVF_RP	GTGGGTCCGAGAGTYTGC
	RVF_P	[FAM]-TCCTTCTCCCAGTCAGCCCCAC-[BBQ]
CCHFV	CCSMW_FP	GGYACYAAGAAAATGAAGAAGG
	CCSMW_RP	CRGGGAKTGTYCCRAAGCA
	CCSMW_LNA	[FAM]-CTTTCCAGSARAAC+A+G+G+AT-[BBQ]

**Table A2:** Mosquito species composition and sex distribution.

Genus	Species	Total (*n*)	Females (♀)	Males (♂)
*Aedes*	*Aedes aegypti*	87	70	17
	*Aedes africanus*	3	3	0
	*Aedes argenteopunctatus*	2	2	0
	*Aedes mcintoshi*	24	24	0
	*Aedes metallicus*	3	3	0
	*Aedes ochraceus*	313	256	57
	*Aedes* spp.	14	14	0
	*Aedes sudanensis*	29	27	2
	*Aedes vexans*	714	657	57
*Anopheles*	*Anopheles gambiae*	34	31	3
	*Anopheles pharoensis*	14	10	4
	*Anopheles rufipes*	16	13	3
	*Anopheles* spp.	32	22	10
	*Anopheles squamosus*	3	3	0
	*Anopheles ziemanni*	16	15	1
*Culex*	*Culex antennatus*	546	369	177
	*Culex bitaeniorhynchus*	32	21	11
	*Culex decens*	20	20	0
	*Culex ethiopicus*	25	25	0
	*Culex neavei*	451	442	9
	*Culex poicilipes*	21	20	1
	*Culex quinquefasciatus*	1144	873	271
	*Culex sitiens*	494	484	10
	*Culex* spp.	48	48	0
	*Culex tritaeniorhynchus*	2441	2052	389
	*Culex univittatus*	32	32	0

**Table A3:** Summary of tick species collected by taxon, sex, and developmental stage.

Genus	Species	Total (*n*)	♀ (*n*)	♂ (*n*)	Nymph (*n*)
*Amblyomma*	*Am.* spp. (undetermined)	6	–	–	6
	*Am. Variegatum*	37	10	27	–
*Hyalomma*	*Hy. Impeltatum*	63	20	43	–
	*Hy. Rufipes*	177	27	144	6
	*Hy.* spp. (undetermined)	4	–	–	4
	*Hy. Truncatum*	65	13	52	–
*Rhipicephalus*	*Rh. evertsi evertsi*	250	74	176	–
	*Rh. Guilhoni*	374	133	241	–
	*Rh. Mushamae*	849	232	613	4
	*Rh.* spp. (undetermined)	121	–	–	121

“♀” and “♂” indicate female and male adult ticks, respectively; “spp.” denotes undetermined species within the genus. Some species include nymphal stages in addition to their adult forms. Dashes (–) indicate that the stage/sex was not applicable or not identified.
